# Failures and lessons learned from the medical school quota expansion policy under the Yoon Suk Yeol administration

**DOI:** 10.3389/fpubh.2026.1971516

**Published:** 2026-10-12

**Authors:** Yu Jin Kim, So Young Kim, Jong Hyock Park

**Affiliations:** 1 Department of Medicine, College of Medicine, Chungbuk National University, Cheongju, Republic of Korea; 2 Institutes of Health and Science Convergence, Chungbuk National University, Cheongju, Republic of Korea; 3 Department of Public Health and Preventive Medicine, Chungbuk National University Hospital, Cheongju, Republic of Korea

**Keywords:** health policy reform, health workforce policy, medical school quota, physician workforce, South Korea

## Abstract

South Korea offers a valuable case for examining why health reforms facing strong physician opposition can produce different outcomes. This commentary compares the 2000 Separation of Drug Prescribing and Dispensing (SPD) with the recent medical school quota expansion using Mark Moore’s public value framework. We assess how public value, the authorizing environment, and operational capacity shaped the trajectories of the two reforms. SPD articulated a clear patient-safety rationale, secured broad political and social authorization, and developed legal, financial, and organizational mechanisms that supported implementation. By contrast, medical school quota expansion combined strong initial public support with contested policy logic, limited stakeholder authorization, and insufficient capacity to expand medical education or direct additional physicians toward underserved specialties and regions. The comparison also shows how earlier policy settlements shaped expectations and bargaining behavior in subsequent reform. Durable health workforce reform therefore requires alignment among public legitimacy, political authorization, and operational capacity.

## Introduction: why this policy failure matters

South Korea offers a valuable case for examining why certain health system reforms withstand organized professional resistance while others fail. The Separation of Drug Prescribing and Dispensing (SPD) in 2000 and the recent medical school quota expansion proposed under the Yoon Suk Yeol administration both aimed to address structural weaknesses in the health system, leading to nationwide physician strikes and major disruptions in care (1, 2). The two reforms produced different outcomes: SPD was implemented and became a lasting institutional change in pharmaceutical governance, whereas the medical school quota expansion encountered continued conflict and policy deadlock.

This contrast highlights a broader challenge in health policy. Although both reforms involved strong state authority and direct challenges to professional interests, they differed in how their goals were translated into public legitimacy and actionable policy. Understanding why two reforms aimed at addressing structural weaknesses in the health system led to different outcomes is crucial, as this insight may inform South Korea and other countries facing physician shortages, regional imbalances, and resistance from organized medical professions (2–4).

This study examines the sources of this divergence using Mark Moore’s public value framework (5). Existing analyses of health workforce and SPD reform largely focus on technical factors, such as workforce projections, or the political influence of professional groups (1, 6, 7). These perspectives provide valuable insights but are less effective in explaining why reforms fail, even when governments possess formal authority and a plausible policy rationale. Moore’s framework emphasizes the conditions necessary for policy durability rather than policy intent alone. It examines whether a reform generates public value, secures a broad and stable authorizing environment, and possesses the operational capacity required for implementation (5). Therefore, this study aims to analyze how the alignment or misalignment of these three elements shaped the trajectories of two major health policy reforms in South Korea.

## Public value as a condition for policy durability

Public value refers to the socially constructed justification for why a policy is necessary and who stands to benefit (5). Even well-designed reforms may fail without a strong, shared rationale to maintain legitimacy during conflict.

For SPD, the public value proposition was clear and broadly appealing. While the legal basis for separation was established in 1963, the issue gained political significance after the 1993 herbal medicine dispute, which reframed it as a matter of public concern (6, 8). By the late 1990s, problems such as antibiotic overuse, irrational prescribing, and conflicts of interest in physician dispensing had framed SPD as a patient safety reform rather than merely a technical regulatory adjustment (6). The phrase “doctors prescribe, pharmacists dispense” condensed this rationale into a clear and publicly resonant narrative. Consequently, even intense physician strikes following the 2000 implementation failed to reverse the reform. This durability reflects the strength of the public value narrative, which was challenging to reject publicly, along with the capacity of the government to manage opposition through negotiated changes.

The public value challenge of medical school quota expansion was more complex than a simple lack of public support. The proposal initially received substantial public support; a national poll conducted shortly after the announcement found that 76% of respondents viewed the expansion positively (9). This support emerged in a context of widespread public concern about highly visible problems in access to care, including repeated emergency department rejections and difficulties securing timely emergency treatment, as well as persistent physician shortages in regional areas and essential specialties (10–12). These problems made physician shortages a salient public concern and formed an important backdrop to the strong initial public support for expansion.

However, broad agreement on the underlying problem did not necessarily translate into consensus on the specific policy instrument or the proposed scale of expansion. The causal logic of the reform remained contested. Expanding the overall physician supply did not directly address the structural causes of maldistribution, payment inequities, specialty avoidance, regional disincentives, and poor working conditions (13). Thus, the weakness in public value lay less in the absence of societal demand for reform than in the limited technical and policy legitimacy linking a uniform increase of 2,000 medical school seats to improvements in essential and regional care. In other words, the reform enjoyed strong legitimacy around the problem but weaker legitimacy around the proposed solution. Unlike SPD, the central disagreement was not whether a health system problem existed, but whether the chosen policy instrument provided a credible mechanism for addressing that problem.

## Authorizing environment and political legitimacy

The authorizing environment encompasses the institutional actors whose support confers legitimacy and enables policy implementation (5). Even reforms that generate public value may fail without sufficiently broad and stable authorization.

For SPD, the authorizing environment was broad, formalized, and institutionally durable. The reform was grounded in legislative amendments to pharmaceutical and health insurance laws, providing a stable statutory basis rather than relying solely on executive discretion (6). Its legitimacy extended beyond the central government to a wider coalition of legislators, health bureaucrats, pharmacists, and civic actors advocating pharmaceutical transparency (14). Authorization was distributed across various institutional actors, making it difficult for intense physician opposition to isolate or delegitimize the reform. While the state faced strong resistance, SPD endured nationwide strikes without policy reversal.

The authorizing environment for medical school quota expansion was not uniformly weak but uneven across different constituencies. Electoral and public authorization was initially strong, as broad public support for increasing the physician workforce provided a substantial public mandate for the reform (9). Stakeholder and institutional authorization, however, was considerably weaker. Although the government possessed formal authority to increase admission quotas, it pursued the reform largely through executive initiative without sufficient prior alignment with key actors responsible for implementation, including medical associations, medical schools, academic leaders, teaching hospitals, and postgraduate training systems (4). This imbalance created an asymmetric authorizing environment: the reform possessed substantial public and electoral authorization but lacked comparable stakeholder and institutional authorization. The top-down policy process reinforced perceptions of unilateral policymaking among professional and educational stakeholders. Physician organizations criticized the proposal as insufficiently evidence-based and misaligned with the structural determinants of workforce maldistribution, including specialty distribution and regional disparities (15, 16).

Additionally, opposition intensified as residents, interns, and medical students joined collective refusals, transforming the dispute from a workforce policy negotiation into a broader governance crisis affecting service delivery and future physician training capacity (9, 14). The 2024 experience therefore demonstrates that broad public support and formal governmental authority may be insufficient when actors whose cooperation is essential to implementation are not incorporated into a durable authorizing coalition.

## Operational capacity and implementation

Operational capacity ultimately determines whether policy intent can be translated into sustained practice (5). Without appropriate institutional arrangements, even reforms backed by public value and political support may fail during implementation.

For SPD, operational capacity was developed through coordinated institutional redesign rather than assumed beforehand. The reform restructured professional roles by separating prescribing and dispensing functions while expanding the role of community pharmacies in medication management (8). This differentiation was reinforced through adjustments to National Health Insurance reimbursement, compensating for shifts in provider income and aligning financial incentives with the new care model (1). Furthermore, the reform embedded enforceable mechanisms, including prescription-based dispensing, reimbursement rules, and regulatory oversight, to ensure compliance in routine clinical practice (1). Although implementation imposed short-term fiscal and administrative strain, these institutional supports enabled the separation of prescribing and dispensing to become institutionalized within the health system.

Conversely, the operational capacity supporting medical school quota expansion was uncertain and underdeveloped. A rapid increase (>60%) in annual medical school admissions requires parallel expansion of faculty, clinical training environments, and residency positions (4). In South Korea, where medical education is closely integrated with teaching hospitals, these elements are interdependent: clinical faculty must balance service delivery, education, and research, while training capacity is constrained due to hospital infrastructure and patient volume (17). However, the expansion policy lacked a clearly articulated plan to scale these components in a coordinated manner. Consequently, concerns emerged about maintaining training quality and clinical supervision during rapid expansion.

More fundamentally, the policy lacked operational mechanisms to align increased physician supply with system needs. In South Korea, workforce imbalances are driven not only by overall supply but also by specialty and regional distribution (18). However, the expansion lacked binding mechanisms, such as targeted placement, incentive structures, and regulatory requirements, to direct graduates toward underserved specialties and regions. Without these mechanisms, increased admissions could reinforce existing concentration patterns rather than correct them (16). Operational capacity therefore required more than expanding educational inputs. It also required downstream mechanisms linking newly trained physicians to areas of need, such as geographically targeted admission pathways, service-linked scholarships, incentives for practice in underserved regions, residency allocation aligned with shortage specialties, and payment reforms that improve the attractiveness of essential care (19). Without such mechanisms, increasing the number of medical graduates could not ensure corresponding improvements in specialty or geographic distribution. From an operational perspective, the issue was not scale alone but alignment: the policy expanded inputs without establishing the necessary institutional pathways for its intended workforce outcomes.

## Path dependence: the legacy of the SPD settlement

The relationship between the two reforms illustrates how earlier policy settlements shape the political feasibility of later reforms. During the 2000 SPD conflict, the government introduced several concessions to physicians to stabilize the reform and restore medical services. These included substantial fee increases and subsequent reductions in medical school admissions (4, 20). Consequently, the national medical school quota declined from 3,507 in the early 2000s to 3,058 by 2006 and remained unchanged for nearly two decades. The prolonged restriction in physician intake, alongside a rapidly aging population and increased demand for medical services, contributed to shortages in specific specialties and regions.

The 2024 medical school quota expansion can be understood as a delayed response to these accumulated pressures. However, the legacy of SPD extended beyond workforce supply by institutionalizing a bargaining template in which collective professional resistance was followed by policy concessions or compensatory measures (7). Repeated experience with such settlements may generate moral-hazard-like incentives: when organized withdrawal from service is perceived to increase the probability of policy modification, rollback, or compensation, professional groups have a strategic rationale for adopting non-cooperative bargaining strategies in subsequent disputes. This dynamic is consistent with a game-theoretic interpretation in which repeated interactions shape expectations about the likely payoffs of cooperation and resistance. In this sense, physician resistance in 2024 reflected not only opposition to the substantive policy but also strategic behavior shaped by prior institutional experience. From a veto-player perspective, repeated policy concessions may also strengthen the effective blocking power of organized professional groups by increasing the credibility of collective resistance as a strategy (21). The resulting deadlock illustrates how earlier policy compromises can evolve into path-dependent bargaining rules, making unilateral policy change increasingly difficult to sustain.

## Policy implications for health workforce reform

The comparison between SPD and medical school quota expansion highlights how alignment across public value, the authorizing environment, and operational capacity shapes reform outcomes (Figure 1). SPD endured not because conflict was absent, but because these conditions aligned more effectively and remained sustained throughout implementation.

**Figure 1:**
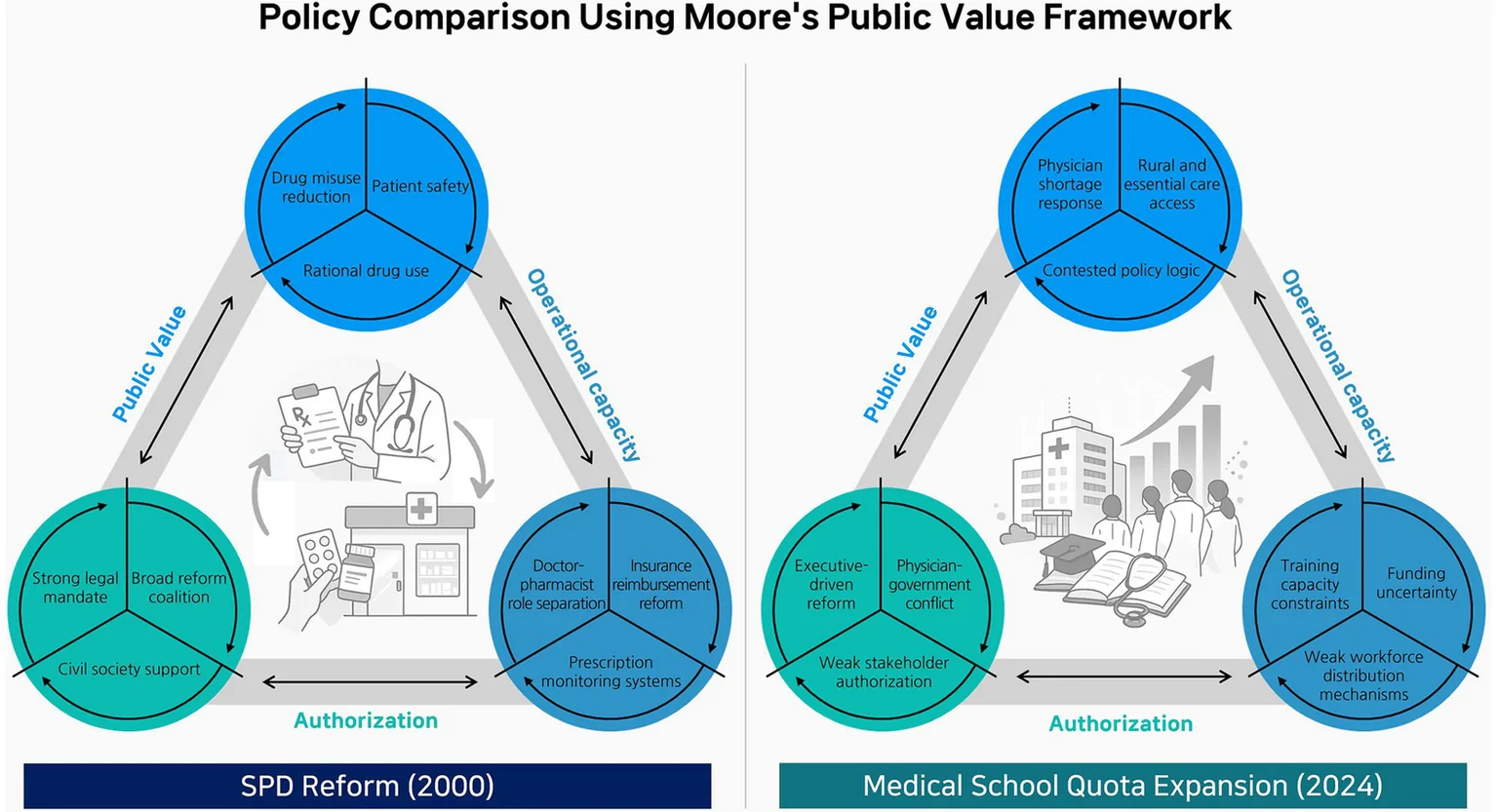
Application of Mark Moore’s public value framework to SPD and medical school quota expansion in South Korea. SPD, Separation of Drug Prescribing and Dispensing.

In contrast, medical school quota expansion showed a more complex pattern of misalignment: strong initial public support but contested policy logic, substantial electoral authorization but weak stakeholder and institutional authorization, and insufficient operational capacity. Three implications emerge from this comparison. First, increasing physician supply alone is unlikely to resolve workforce maldistribution without complementary policies that address underlying structural drivers, including payment systems, working conditions, and career incentives. Without these measures, expanded physician supply may reinforce existing concentration patterns rather than improve access in underserved specialties and regions. Second, broad public support for a policy goal should not be equated with legitimacy of a specific policy instrument. Reform durability depends on establishing a credible causal link between the public value being pursued and the mechanisms selected to achieve it. Public concern about physician shortages may provide political authorization for reform, but it does not by itself establish that a particular quota increase will improve essential or regional care. Third, implementation feasibility depends on the strength of the authorizing environment and the adequacy of operational capacity. Formal governmental authority and electoral support must be complemented by institutional arrangements that secure cooperation from actors responsible for medical education, training, and service delivery. Reforms lacking broad institutional support or mechanisms to align workforce distribution with system needs are unlikely to achieve sustained influence, even when their goals are widely recognized.

These lessons extend beyond South Korea. Health systems facing physician shortages frequently confront a similar tension between increasing aggregate workforce supply and addressing where and in which specialties physicians actually practice. Japan, for example, has used regional admission pathways and related workforce policies to link medical education more directly to geographic distribution, illustrating that expansion of training capacity can be combined with mechanisms targeting underserved areas (19, 22). Professional authorization is equally important. Recent junior doctor industrial action in England similarly illustrates how prolonged disputes between governments and medical professionals can disrupt health services and require sustained negotiation to reach policy settlements (23). Although institutional contexts differ across countries, these cases reinforce a common lesson: increasing physician numbers is unlikely to be sufficient unless governments simultaneously address workforce distribution, implementation capacity, and institutionalized channels for professional negotiation.

## Conclusion

Recent policy developments in South Korea suggest recalibration rather than abandonment. Following the enactment of the Special Act on Strengthening Essential Health Care Support and Reducing Regional Health Care Gaps and subsequent policy adjustments, the reform trajectory has shifted toward a more negotiated and incremental approach, combining gradual expansion of medical school admissions with efforts to rebuild political and professional consensus (24, 25). In this respect, the evolving trajectory of medical school quota expansion appears to be moving gradually toward the conditions outlined in Moore’s public value framework. Although this analysis focuses on South Korea, it may provide valuable insights for other health systems facing similar workforce reform challenges amid strong professional influence and implementation constraints.

## Data Availability

The original contributions presented in the study are included in the article/supplementary material, further inquiries can be directed to the corresponding author.
